# Retention of female volunteer community health workers in Dhaka urban slums: a prospective cohort study

**DOI:** 10.1186/1478-4491-12-29

**Published:** 2014-05-20

**Authors:** Khurshid Alam, Elizabeth Oliveras

**Affiliations:** 1 Equity and Health Systems, International Centre for Diarrhoeal Disease Research, Bangladesh (ICDDR, B), 68 Shaheed Tajuddin Ahmed Sharani, Mohakhali, Dhaka 1212, Bangladesh; 2 Monash School of Public Health & Preventive Medicine, Monash University, 99 Commercial Road, Level 5, The Alfred Centre, Melbourne, VIC 3004, Australia; 3 FHI 360, Rua dos Sinais No 50/74, Maputo, Mozambique

**Keywords:** Community health service delivery, Community health workers, Volunteer retention, Urban slums, Dhaka

## Abstract

**Background:**

Volunteer community health workers (CHWs) are a key approach to improving community-based maternal and child health services in developing countries. BRAC, a large Bangladeshi non-governmental organization (NGO), has employed female volunteer CHWs in its community-based health programs since 1977, recently including its *Manoshi* project, a community-based maternal and child health intervention in the urban slums of Bangladesh. A case–control study conducted in response to high dropout rates in the first year of the project showed that financial incentives, social prestige, community approval and household responsibilities were related to early retention in the project. In our present prospective cohort study, we aimed to better understand the factors associated with retention of volunteer CHWs once the project was more mature.

**Methods:**

We used a prospective cohort study design to examine the factors affecting retention of volunteer CHWs who remained in the project after the initial start-up period. We surveyed a random sample of 542 CHWs who were working for BRAC *Manoshi* in December 2008. In December 2009, we revisited this cohort of CHWs and interviewed those who had dropped out about the main reasons for their dropping out. We used a multivariable generalized linear model regression analysis with a log link to estimate the relative risk (RR) of independent factors on retention.

**Results:**

Of the 542 CHWs originally enrolled, 120 had dropped out by the end of one year, mainly because they left the slums. CHWs who received positive community appraisal (adjusted RR = 1.45, 95% confidence interval (CI) = 1.10 to 1.91) or were associated with other NGOs (adjusted RR = 1.13, 95% CI = 1.04 to 1.23) were more likely to have been retained in the project. Although refresher training was also associated with increased retention (adjusted RR = 2.25, 95% CI = 1.08 to 4.71) in this study, too few CHWs had not attended refresher training regularly to make it a meaningful predictor of retention that could be applied in the project setting.

**Conclusion:**

Factors that affect retention of CHWs may change over time, with some factors that are important in the early years of a project losing importance as the project matures. Community health programs operating in fragile urban slums should consider changing factors over program duration for better retention of volunteer CHWs.

## Background

In the past decade, deployment of community health workers (CHWs) has been promoted around the world as a means of both addressing the healthcare workforce crisis and meeting the Millennium Development Goals by 2015 [1-3]. These workers are seen as the best means by which to reach underserved populations, particularly in remote and underprivileged communities, whose needs are not met by their existing local healthcare system. However, high dropout rates hamper the success of volunteer-based programs [4], thus reducing program stability and increasing training costs due to the continuous need for replacement [1]. Dropout of volunteer CHWs is defined as the decline in the pool of eligible, trained CHWs who are expected to continue in the project until the project is completed. Identifying predictors of retention and dropout may help program managers to strengthen their selection processes, modify the incentives and support they provide for CHWs and highlight other factors that they might try to influence in order to improve CHW retention.

Studies of volunteers in these settings show that a range of factors can affect retention and dropout. Retention and attrition have been found to be related to financial incentives [5-12], community approval or disapproval [8,10,11,13-15], familial approval or disapproval [12,13], the potential value of the CHW position in securing future career advancement [12], dissatisfaction with pay [12], heavy workload [12], night visits [12], supportive supervision and achievement of personal growth through training and practice [16,17].

The majority of these previous studies were conducted in rural areas. In the only study of urban CHWs, a small study in one urban slum, competition from other sources of employment was an additional cause of dropout that had not previously been identified in rural sites [14]. The local labour market and local livelihood competition appear to be different in urban areas than in rural areas and may also affect CHW retention and job performance.

### Community health workers in urban slums of Bangladesh

BRAC, a large Bangladeshi non-governmental organization (NGO), pioneered the use of female volunteer CHWs (popularly known as *Shasthya Shebika*) beginning in the 1970s. BRAC recruits and trains female volunteer CHWs who serve as the first point of contact between community members and BRAC, which provides essential healthcare services [18]. Currently, about 80,000 female volunteer CHWs work throughout Bangladesh in BRAC healthcare programs both in rural and urban settings. BRAC introduced volunteer CHWs into urban slums in the mid-2000s in a maternal, newborn and child health project called *Manoshi*.

BRAC *Manoshi* recruited female volunteer CHWs from the project communities. CHWs had to be members of a BRAC village organization (VO), over 25 years old, married but without a child under two years of age, interested in serving as a volunteer and acceptable to the community. The project did recruit non-VO members if suitable VO members were not available in a community. Each branch office conducted a needs assessment survey in the respective catchment area and identified prospective candidates from the community for the volunteer CHW positions. Final selection of CHWs was held at the branch office under the active supervision of the respective Branch Manager. In *Manoshi*, each CHW was responsible for overseeing an average of 200 households and visiting 8 to 10 of them per day. They visited homes to disseminate healthcare messages, identified pregnancies, brought pregnant women to delivery centres, accompanied pregnant women during their delivery and provided newborn care.

Although BRAC’s CHWs are volunteers, they often see their role as profit-making because they receive performance-based financial incentives from BRAC for their work [9]. *Manoshi* CHWs received financial incentives for pregnancy identification, bringing pregnant women to *Manoshi* delivery centres and attending to mothers and newborns after delivery [15,19,20]. They were also able to make some money by selling drugs and BRAC’s health commodities, and they received an allowance for attending a refresher training course each month.

An earlier investigation of CHW retention in the first two years of the project was done using a case–control study design to identify factors that might improve retention [15]. In the present study, we build on the results of that investigation with the use of a prospective cohort study design to gather evidence about any long-term effects of previously identified retention factors and to determine whether there are any new factors associated with retention.

## Methods

We employed a prospective cohort study design to examine factors associated with retention of volunteer CHWs working for BRAC’s *Manoshi* project. We measured potential predictors of retention at enrolment, although we interviewed dropped out CHWs at one-year follow-up regarding their primary reasons for dropping out. We conducted the study in 12 *Manoshi* branch offices that had been in operation for more than two years at the time of enrolment. The Institutional Review Board of the International Centre for Diarrheal Disease Research, Bangladesh (ICDDR, B) approved the study protocol.

### Sample

**Figure 1:**
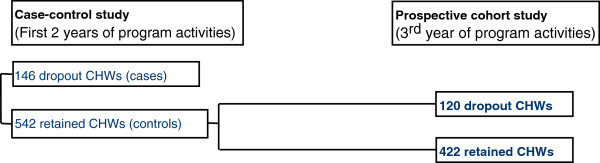
Flowchart on selection of sample.

For the case–control study on retention during the first two years of the project, a simple random sample of 542 CHWs from the population of 1,125 current CHWs listed in the *Manoshi* registers at the time of the study was selected [15]. We nested this prospective cohort study within the original case–control study. The controls were from that case–control study, those CHWs who were current (that is, retained), formed the cohort population in this study (Figure 1).

For the initial retention study, the Epi Info software (http://www.who.int/chp/steps/resources/EpiInfo/en/) of the World Health Organization was used to estimate the required sample size assuming an unmatched case–control design. The study estimated the proportion of controls who were exposed to factors of interest (difficulty in educating children and fears and misconceptions of family members about the BRAC health program) at 15%, based on an existing study of BRAC urban CHWs [14]. In addition, the study assumed that the odds ratio of dropping out associated with exposure was 2 at the 95% confidence level and 80% power. For a ratio of one case to four controls, the number of cases required was at least 133 dropout CHWs. The study sampled both current CHWs and dropout CHWs using project data and ended up with a total sample of 146 dropout CHWs and 542 current CHWs who participated in the survey. The detailed sampling strategy is also available elsewhere [15,20].

### Eligibility criteria

As in the original case–control study, we determined eligibility for this study based on BRAC’s criteria for the *Manoshi* project. We considered a woman who had completed a three-week basic training course to be a volunteer CHW and considered her to be current if she was in the *Manoshi* register at the time of study enrolment. *Manoshi* Branch Managers updated registers regularly on the basis of the availability of CHWs to perform assigned activities and attendance at monthly refresher training sessions.

### Data collection

Baseline data were collected on potential factors related to retention in December 2008. Four trained field research staff interviewed the sampled volunteer CHWs using a pre-tested structured questionnaire after obtaining the CHW’s written informed consent. After one year of follow-up, in December 2009, we again used the BRAC registers to identify CHWs who had dropped out during the follow-up period. We then recontacted and interviewed these dropout CHWs about their primary reasons for dropping out. We collected no additional data on factors related to retention during the follow-up interview.

### Outcome measure

We considered CHWs to be retained if they were still in the BRAC *Manoshi* registers at the one-year follow-up interview (1 if the CHW was continuing with the program or 0 if the CHW had dropped out).

### Measures of independent variables

**Figure 2:**
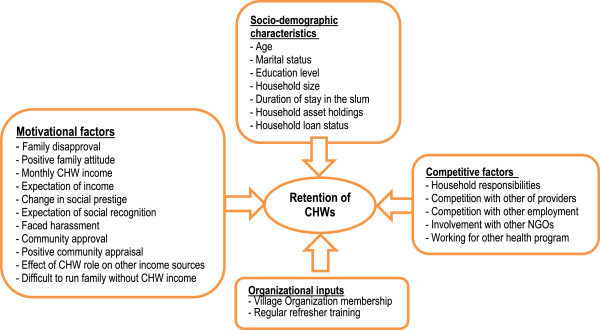
**Conceptual framework of factors affecting retention of volunteer community health workers, Dhaka urban slums, 2009.**

We identified potential predictors of retention of volunteer CHWs based on a review of the literature and input from BRAC *Manoshi* project staff. Furthermore, because the use of CHWs in urban slums is rare, we hypothesized, together with project staff, additional factors that might be particular to this environment, such as competition from other healthcare providers and competition from alternative employment. We classified the identified characteristics into four categories: sociodemographic characteristics*,* motivational factors*,* organizational inputs and competitive factors (Figure 2).

*Sociodemographic characteristics* included age (<25 years, 25–45 years and >45 years), marital status (unmarried, married and widowed, divorced or separated), educational attainment (whether the CHW had completed grade five or higher), household size, duration of stay in slum (<10 years, 10 to 20 years and >20 years), household asset quintiles (derived by applying principal component analysis) and whether the household of the CHW had an outstanding loan.

*Motivational factors* were factors that might encourage or discourage the volunteer CHWs to continue with the BRAC *Manoshi* project. They included whether the CHW experienced family disapproval, whether her family had a positive attitude towards her CHW role, self-reported average monthly CHW income (<US$ 5.92, US$ 5.92 to US$ 7.64 and > US$ 7.64), whether she had joined BRAC expecting income from her CHW work, changes in social prestige (more than before, no change and less than before—derived using factor analysis of whether the CHW received social invitations, positive greetings in the street, informal credit or invitations to resolve familial or social disputes), whether she had joined BRAC expecting social recognition, whether she faced harassment in the community, whether the community approved of the CHW role, whether she received positive community appraisal, whether her CHW role positively affected earnings from other income sources and whether she faced difficulty in supporting her family without CHW income.

*Organizational inputs* were direct inputs provided by BRAC. They included VO membership (whether the CHW was involved with the BRAC microfinance group) and whether she attended refresher training every month.

*Competitive factors* were factors that might compete with her performance or earnings. They included whether the CHW experienced conflicts with household responsibilities, whether she experienced competition with other providers, whether she was involved in other income-earning activities (competition with other employment), whether she was involved with other NGOs in a non-healthcare-related role and whether she worked for another health program and/or hospital in a healthcare worker role.

### Data analysis

We conducted χ^2^ tests for proportions to examine the percentage of CHWs retained at each level of the categorical independent variables measured at enrolment. Prior to modelling, we tested for multicollinearity between all potential independent variables using variance inflation factors and found no evidence of multicollinearity. We calculated unadjusted relative risk (RR) and 95% CI for all exposure variables. We carried out multivariable generalized linear model regression analysis with a log link to estimate the RR of the independent variables on retention of volunteer CHWs. To identify covariates for the multivariable model, we created a series of multivariable models in which we sequentially added a set of variables representing each of the four categories of explanatory factors (Figure 2) to the model in chunkwise regression [21]. We excluded variables that were not significant (*P* > 0.05) or not confounders and which we did not consider theoretically important [22]. However, we kept the variables that we had found to be significantly associated with CHW retention in the previous case–control study [15], regardless of their statistical significance in the current analysis. In this way, we reached the most parsimonious model for identifying the final explanatory factors likely affecting retention of volunteer CHWs. We conducted all analyses using STATA 12.1 software (StataCorp, College Station TX, USA).

## Results

All of the 542 CHWs sampled agreed to participate in the study, and we interviewed them. At the time the study began, the mean age of these CHWs was 32.3 years, 86.2% were currently married, the average family size was 4.7 persons, the monthly average CHW income was US$ 8.15 and 41.5% had completed primary education or higher.

At one year of follow-up 120 CHWs (22%) had dropped out. All of them participated in the reinterview. The main reasons that CHWs gave for dropping out of the *Manoshi* project are listed in Table 1. Fifty-four (45%) of the dropout CHWs had left the project because they had left the slum. Eighteen (15%) of them had discontinued because there was no regular salary as a volunteer and another 15% had discontinued because of a conflict with family time. Another 13 CHWs (11%) discontinued because of disapproval of their husband or family members.

**Table 1:** **Primary reasons for dropout of volunteer community health workers**, **Dhaka urban slums**, **2009**

**Reason for dropping out**	***n***	**(%)**
Left slums	54	(45.00)
No salary	18	(15.00)
Time conflict	18	(15.00)
Disapproval from husband and family	13	(10.83)
Slum evictions	5	(4.17)
Old age and sickness	4	(3.33)
BRAC terminated	4	(3.33)
Others (became birth attendant, misbehaviour of BRAC staff, do not like the job)	4	(3.33)
Total	120	(100)

Table 2 shows the percentage of CHWs retained at each level of the categorical independent predictors that we hypothesized might be related to retention. In these univariate analyses, a number of sociodemographic variables were associated with retention. In particular, a larger percentage of CHWs 25 to 45 years of age was retained compared to CHWs younger than 25 years of age (80.2% vs 68%, unadjusted RR = 1.18). Length of stay in the slum was also related, with a higher rate of retention among CHWs who stayed in the slum >10 years compared to those who stayed <10 years (more than 80% were retained among those who stayed either 10 to 20 years or >20 years vs 70.7% among those who stayed <10 years). A higher percentage of CHWs in the lower, middle and richest quintiles were retained than those in the poorest quintile (83.7% in the lower middle and 81.7% in the richest quintile vs 71.6% in the poorest quintile). CHWs with the highest average monthly income from CHW work were more likely to be retained compared to those CHWs with the lowest average monthly income (82.6% vs 73.6%, unadjusted RR = 1.12). With regard to motivational factors, the percentage of CHWs retained was higher among those CHWs who received positive community appraisal than among those who did not (79.5% vs 56.4%, unadjusted RR = 1.41) and also was higher among those who were associated with another NGO than among those who were not (83.5% vs 73.1%, unadjusted RR = 1.14). At the organizational level, CHWs who regularly attended refresher training were also more likely to be retained (79.1% vs 33.3%, unadjusted RR = 2.37), but very few CHWs did not attend regularly (*n* = 15).

**Table 2:** **Univariate analysis of retained volunteer community health workers by level of categorical predictors**, **Dhaka urban slums**, **2009**^**a**^

**Independent variables**	**Retained CHWs (%)**	**RR (95% CI)**
	**(1)**	**(2)**
**Sociodemographic characteristics**		
Age		
>25 yr of age (*n* = 103)	67.96	1.00
25 to 45 yr of age (*n* = 394)	80.20	1.18^b^ (1.02 to 1.36)
>45 yr of age (*n* = 45)	80.00	1.18 (0.97 to 1.43)
Marital status		
Unmarried (*n* = 11)	54.55	0.70 (0.41 to 1.20)
Married (*n* = 467)	78.16	1.00
Widow, divorced or separated (*n* = 64)	79.69	1.02 (0.89 to 1.16)
Primary education or higher		
Yes (*n* = 225)	74.67	0.93 (0.85 to 1.02)
No (*n* = 317)	80.13	
Duration of stay in the slum		
*<*10 yr (n *=* 167)	70.66	1.00
10 to 20 yr (*n* = 213)	80.28	1.14^b^ (1.01 to 1.28)
>20 yr (*n* = 162)	82.10	1.16^b^ (1.03 to 1.31)
Household asset quintiles		
Poorest (*n* = 116)	71.55	1.00
Lower middle (*n* = 104)	83.65	1.17^b^ (1.01 to 1.35)
Middle (*n* = 120)	75.00	1.05 (0.90 to 1.22)
Upper middle (*n* = 82)	78.05	1.09 (0.93 to 1.28)
Richest (*n* = 120)	81.67	1.14^c^ (0.99 to 1.32)
Household had a loan		
Yes (*n* = 232)	81.03	1.07 (0.98 to 1.17)
No (*n* = 310)	75.48	
**Motivational factors**		
Family disapproval		
Yes (*n* = 150)	80.67	1.05 (0.95 to 1.16)
No (*n* = 392)	76.79	
Positive family attitude		
Yes (*n* = 441)	78.23	1.03 (0.91 to 1.16)
No (*n* = 101)	76.24	
Average monthly CHW income		
<US$ 5.92 (*n* = 231)	73.59	1.00
US$ 5.92 to US$ 7.64 (*n* = 99)	77.78	1.06 (0.93 to 1.20)
>US$ 7.64 (*n* = 212)	82.55	1.12^b^ (1.02 to 1.24)
Joined with expectation of income		
Yes (*n* = 328)	78.35	1.02 (0.93 to 1.11)
No (*n* = 214)	77.10	
Change in social prestige		
Less than before (*n* = 141)	73.05	0.91 (0.81 to 1.03)
No change (*n* = 203)	80.30	1.00
More than before (*n* = 198)	78.79	0.98 (0.89 to 1.08)
Joined with expectation of social recognition		
Yes (*n* = 150)	74.00	0.93 (0.84 to 1.04)
No (n = 392)	79.34	
Harassed while working in the community		
Yes (*n* = *64*)	85.94	1.12^b^ (1.00 to 1.25)
No (*n* = 478)	76.78	
Community approval		
Yes (*n* = 427)	78.22	1.02 (0.91 to 1.14)
No (*n* = 115)	76.52	
Positive community appraisal		
Yes (*n* = 503)	79.52	1.41^b^ (1.07 to 1.86)
No (*n* = 39)	56.41	
Effect of CHW role on other income sources		
Yes (*n* = 53)	69.81	0.89 (0.74 to 1.06)
No (*n* = 489)	78.73	
Difficult to run family without CHW income		
Yes (*n* = 306)	78.10	1.01 (0.92 to 1.10)
No (*n* = 236)	77.54	
**Organizational inputs**		
Village organization membership		
Yes (*n* = 190)	79.47	1.03 (0.94 to 1.13)
No (*n* = 352)	76.99	
Attending refresher training regularly		
Yes (*n* = 527)	79.13	2.37^b^ (1.16 to 4.87)
No (*n* = 15)	33.33	
**Competitive factors**		
Conflict with household responsibilities		
Yes (*n* = 74)	78.38	1.01 (0.89 to 1.15)
No (*n* = 468)	77.78	
Competition with other providers		
Yes (*n* = 60)	78.22	1.04 (0.89 to 1.22)
No (*n* = 482)	75.00	
Competition with other employment		
Yes (*n* = 208)	79.33	1.03 (0.94 to 1.13)
No (*n* = 334)	76.95	
Involved with other NGOs		
Yes (*n* = 248)	83.47	1.14^b^ (1.04 to 1.25)
No (*n* = 294)	73.13	
Working for another health program		
Yes (*n* = 34)	67.65	0.86 (0.68 to 1.09)
No (*n* = 508)	78.54	

^a^CHW, Community health worker; CI, Confidence interval; NGO, Non-governmental organization; RR, Relative risk. ^b^Significant at the 5%** level. ^c^Significant at the 10%* level. US$ 1.00 = 67.52 Bangladeshi Taka (on 1 July 2008).

After controlling for potential confounders, particularly age, education, marital status, household size, household asset holdings, duration of stay in slums and VO membership, no single group of factors had a notable effect on the retention of volunteer CHWs. However, positive community appraisal, regular attendance in refresher training and involvement with other NGOs were all independently associated with retention (Table 3). The relative risk of retention was 45% higher among those who experienced positive community appraisal than among those who did not (adjusted RR = 1.45, 95% CI = 1.10 to 1.91). The relative risk of retention among CHWs who attended refresher training on a regular basis was more than twice that of CHWs who did not (adjusted RR = 2.25, 95% CI = 1.08 to 4.71). Furthermore, the relative risk of retention of CHWs who were involved with other NGOs was 13% higher than those who did not (adjusted RR = 1.13, 95% CI = 1.04 to 1.23). Counterintuitively, those CHWs whose families disapproved of their CHW role also had a higher relative risk of retention (adjusted RR = 1.12, 95% CI = 1.01 to 1.24). Most factors that were significant predictors of retention during the first two years of the project were not predictors of retention in the current analysis. In particular, household asset holdings, financial incentives, changes in social prestige, expectation of social recognition, community approval and conflict with household responsibilities were not significantly associated with retention in the current model.

**Table 3:** **Risk factors for retention of a cohort of 542 volunteer community health workers, Dhaka urban slums, 2009**^**a**^

**Independent variables**	**Model 1: Sociodemographic characteristics**	**Model 2: Addition of motivational factors**	**Model 3: Addition of organizational inputs**	**Model 4: Addition of competitive factors**	**Model 5: Final model**
	**Partially adjusted RR (95% CI)**	**Partially adjusted RR (95% CI)**	**Partially adjusted RR (95% CI)**	**Partially adjusted RR (95% CI)**	**Adjusted RR (95% CI)**
	**(1)**	**(2)**	**(3)**	**(4)**	**(5)**
**Sociodemographic characteristics**					
Age					
>25 yr of age	1.00	1.00	1.00	1.00	
25-45 yr of age	1.08 (0.92 to 1.27)	1.08 (0.93 to 1.27)	1.09 (0.93 to 1.28)	1.06 (0.91 to 1.25)	
*>*45 yr of age	1.06 (0.85 to 1.32)	1.06 (0.84 to 1.32)	1.05 (0.84 to 1.31)	1.04 (0.83 to 1.30)	
Marital status					
Unmarried	0.74 (0.42 to 1.30)	0.80 (0.46 to 1.38)	0.88 (0.54 to 1.43)	0.85 (0.52 to 1.40)	
Married	1.00	1.00	1.00	1.00	
Widow, divorced, or separated	1.02 (0.89 to 1.16)	1.02 (0.89 to 1.18)	1.02 (0.88 to 1.18)	1.02 (0.88 to 1.18)	
Primary education complete or above	0.97 (0.87 to 1.07)	0.97 (0.87 to 1.08)	0.96 (0.87 to 1.07)	0.96 (0.86 to 1.07)	
Household size	1.02 (0.99 to 1.04)	1.01 (0.99 to 1.04)	1.01 (0.99 to 1.04)	1.02 (0.99 to 1.04)	
Duration of stay in the slum	1.00	1.00	1.00	1.00	1.00
<10 yr	1.11^c^ (0.98 to 1.25)	1.10 (0.98 to 1.24)	1.08 (0.96 to 1.22)	1.07 (0.95 to 1.20)	1.08 (0.96 to 1.21)
10-20 yr	1.12^c^ (0.98 to 1.26)	1.11^c^ (0.98 to 1.25)	1.10 (0.97 to 1.24)	1.09 (0.96 to 1.23)	1.11^c^ (0.99 to 1.25)
>20 yr					
Household asset quintiles	1.00	1.00	1.00	1.00	1.00
Poorest	1.16^b^ (1.01 to 1.33)	1.14^c^ (0.99 to 1.31)	1.15^b^ (1.00 to 1.32)	1.13^c^ (0.98 to 1.29)	1.13^c^ (0.99 to 1.29)
Lower middle	1.05 (0.90 to 1.23)	1.06 (0.91 to 1.23)	1.07 (0.92 to 1.25)	1.07 (0.92 to 1.24)	1.05 (0.91 to 1.22)
Middle	1.08 (0.92 to 1.26)	1.06 (0.90 to 1.25)	1.06 (0.91 to 1.25)	1.05 (0.90 to 1.23)	1.04 (0.89 to 1.22)
Upper middle	1.12 (0.97 to 1.31)	1.10 (0.94 to 1.28)	1.12 (0.96 to 1.29)	1.12 (0.97 to 1.30)	1.12^c^ (0.98 to 1.28)
Richest					
Household loan status	1.04 (0.95 to 1.13)	1.01 (0.93 to 1.10)	1.00 (0.92 to 1.09)	0.95 (0.86 to 1.04)	
**Motivational factors**					
Family disapproval		1.10^c^ (1.00 to 1.22)	1.10^c^ (0.99 to 1.21)	1.12^b^ (1.01 to 1.25)	1.12^b^ (1.01 to 1.24)
Positive family attitude		0.98 (0.87 to 1.11)	0.99 (0.87 to 1.12)	0.99 (0.87 to 1.11)	
Monthly CHW income					
<US$ 5.92		1.00	1.00	1.00	
US$ 5.92 to US$ 7.64		1.03 (0.90 to 1.17)	1.02 (0.89 to 1.16)	1.01 (0.88 to 1.15)	
>US$ 7.64		1.09^c^ (0.99 to 1.20)	1.07 (0.97 to 1.18)	1.06 (0.96 to 1.17)	
Joined with expectation of income		1.02 (0.93 to 1.12)	1.02 (0.93 to 1.12)	1.02 (0.93 to 1.12)	1.04 (0.95 to 1.14)
Change in social prestige		0.90^c^ (0.80 to 1.02)	0.91 (0.80 to 1.02)	0.90^c^ (0.80 to 1.02)	1.0
Less than before		1.00	1.00	1.00	0.91^c^ (0.81 to 1.02)
No change		0.95 (0.86 to 1.05)	0.93 (0.84 to 1.03)	0.94 (0.85 to 1.04)	0.95 (0.86 to 1.05)
More than before					
Joined with expectation of social recognition		0.92 (0.82 to 1.02)	0.92 (0.82 to 1.03)	0.93 (0.83 to 1.03)	0.94 (0.85 to 1.04)
Harassed while working in the community		1.12^c^ (1.00 to 1.26)	1.11^c^ (0.98 to 1.24)	1.08 (0.96 to 1.21)	
Community approval		1.02 (0.91 to 1.15)	1.02 (0.91 to 1.14)	1.01 (0.90 to 1.14)	1.00 (0.89 to 1.11)
Positive community appraisal		1.39^b^ (1.05 to 1.84)	1.39^b^ (1.06 to 1.83)	1.42^b^ (1.08 to 1.87)	1.45^b^ (1.10 to 1.91)
Effect of CHW role on other income sources		0.88 (0.73 to 1.05)	0.91 (0.76 to 1.08)	0.85^c^ (0.70 to 1.02)	0.85^c^ (0.71 to 1.03)
Difficult to run family without CHW income		0.97 (0.88 to 1.07)	0.98 (0.89 to 1.08)	1.01 (0.91 to 1.12)	
**Organizational inputs**					
Village organization membership			1.00 (0.91 to 1.10)	0.95 (0.86 to 1.05)	
Attending refresher training regularly			2.13^b^ (1.03 to 4.39)	2.17^b^ (1.04 to 4.52)	2.25^b^ (1.08 to 4.71)
**Competitive factors**					
Conflict with household responsibilities				0.98 (0.85 to 1.12)	0.98 (0.86 to 1.13)
Competition with other providers				1.01 (0.87 to 1.18)	1.01 (0.88 to 1.16)
Competition with other employment				1.09^c^ (1.00 to 1.20)	1.08^c^ (0.99 to 1.18)
Involvement with other NGOs				1.15^b^ (1.03 to 1.28)	1.13^b^ (1.04 to 1.23)
Working for other health program				0.80^c^ (0.63 to 1.01)	0.81^c^ (0.64 to 1.02)

^a^CHW, Community health worker; CI, Confidence interval; NGO, Non-governmental organization; RR, Relative risk. ^b^Significant at the 5%** level. ^c^Significant at the 10%* level. US$ 1.00 = 67.52 Bangladeshi Taka (on 1 July 2008).

On the whole, competition did not play a key role in retention. Although involvement with other NGOs was associated with increased risk of retention, neither competition with other providers nor competition with other employment was associated with retention at the 5% level of significance. In contrast to our expectations, both were positively rather than negatively associated with retention, particularly with regard to competition with other employment, which was positively associated with retention at the 10% level of significance.

## Discussion

The retention rate of CHWs in the third year of BRAC’s urban *Manoshi* project was nearly 80%, which is somewhat lower than that found in health programs operated by BRAC in rural areas, where the retention rate is 88% [23]. Almost half of all CHWs who dropped out in the third year (45%) reported that they did so because they left their slums. The transitory nature of urban slum dwellers is a unique feature of this environment that is likely to pose a threat for any volunteer program that operates there [24,25]. The fact that this was the leading cause of dropout in our present study may be one reason why few other factors stood out as strong predictors of dropout. Leaving slums is not likely to be associated with other factors explored in this study.

Previously, BRAC researchers found that poor retention of CHWs was related to inappropriate CHW selection, not enough income to sustain work, competing priorities with work at home and adverse sentiments from the community [26]. In our prior analysis of retention of *Manoshi* CHWs in the first two years of the project, we found that expectations regarding income and social recognition, changes in social prestige, wealth quintile, household responsibilities, competition with other providers and community approval were significantly associated with retention of *Manoshi* CHWs [15].

This prospective cohort study allowed us to assess factors associated with retention among those CHWs who remained in the project after two years, presumably those CHWs in whom BRAC had invested more and who were more established in the communities in which they worked. Among the factors important in the first two years of the project, none were significant in this analysis. That being said, the role of the response of the community remained important.

In our prior study [15], community approval of the CHW was a significant predictor of retention, whereas in our present study, those CHWs who received positive appraisal of their personal work from the community had a 45% greater “risk” of retention than those who did not. Although one should interpret with caution the CHWs’ perception of how the community evaluated their role, this finding suggests that positive appraisal from the community can affect CHWs’ performance and motivation. The continued importance of community response, be it to the role of the CHW or to the performance of individual CHW, suggests a need for projects such as BRAC *Manoshi* to work with communities to ensure that the CHW role is properly understood and that volunteers are provided with positive recognition for their work. For example, volunteer health workers in Ethiopia said that an event organized to thank them in front of the community would strengthen their motivation [27].

The lack of an association between household responsibilities and retention may be the result of selection, because women who faced competition from household responsibilities were more likely to drop out in the first two years, and this cohort likely included women who were already less likely to experience such conflict. However, it is notable that when CHWs who dropped out were asked about their primary reasons for doing so, time conflict was among the top three reasons. This apparent discrepancy in the findings merits further exploration, but it may simply be a result of the high proportion who dropped out because they moved out of the slum, as noted above.

Volunteer CHWs who attended refresher training regularly had almost twice the chance of being retained in the program as those who did not. Although refresher training is intended to help CHWs develop skills and confidence in their role, and thereby motivate them to work as CHWs, almost all CHWs had attended refresher training regularly (only 15 of 542 had not), so there is little room for improvement in that aspect. That being said, given the strong effect of regular attendance in refresher training on retention, lack of attendance can serve as a signal of potential dropout. If program managers note that a CHW has stopped attending refresher training regularly, they could intervene to either ensure retention of the CHW or more quickly remove one who is not performing well. In addition, in other programs where attendance is not as close to universal, greater attention to refresher training may be warranted.

CHWs who were involved with other NGOs were significantly more likely to be retained in the *Manoshi* project. Because BRAC CHWs are volunteers rather than full-time employees, they have opportunities to join other NGO programs, such as microfinance, women’s empowerment, informal education and community mobilization programs. Being involved in these activities may indicate a higher level of motivation than that found among their counterparts who are not involved with other NGOs. It is also possible that participation in other NGO programs helps CHWs to develop social networks, linking them to women and concerned stakeholders in the community and facilitating their role as CHWs. Program managers could take this into account in both selecting CHWs and working with them in order to improve retention.

The finding that CHWs who experienced disapproval from their families were more likely to be retained is counterintuitive. We anticipated that CHWs who experienced disapproval would be more likely to drop out. The reason for this discrepancy is unclear and merits further exploration.

The overall study findings need to be interpreted keeping in mind that the data were collected from self-reported activities, which could also have introduced bias. Respondents tend to provide socially acceptable answers or answers that reflect well on them. When they are asked for specific frequencies or amounts, they may rely on best estimates rather than carefully recalling and counting [28]. Current CHWs may be more prone to answering in a manner that they think will improve their chances of remaining as CHWs, and they may report more accurately details about aspects of their experience such as monthly income because they are part of their day-to-day lives.

The results of this study also should be interpreted keeping in mind that potential predictors were measured only at baseline. It is possible that some of these factors changed over the course of the follow-up period and that such changes affected the results. Unfortunately, it is not possible to predict the direction or magnitude of such an effect. Because of the exploratory nature of this study, more than 20 independent variables were considered in the univariate analysis. Although this level of multiple comparisons increases the chance of finding a variable associated with retention as a result of chance alone, the consistency of these findings across the models suggests that the factors identified are robust predictors of retention.

## Conclusions

In this prospective cohort study conducted during year three of the *Manoshi* project in Dhaka urban slums, we identified factors associated with retention of CHWs that are different from those discovered in a prior study of the same project in which retention during the first two years of the project was assessed. These differences may reflect a change over time in the factors associated with retention, particularly early in the life of a project. If this is the case, program managers need to take it into account in implementing strategies to retain CHWs, varying their strategies over time. The one consistent finding is that community reactions influence CHW retention. In this study, community appraisal of the individual affected retention, whereas in our prior study, it was community approval of the CHW role that affected retention. Regardless, this finding points to a need to strengthen efforts to build community support for CHWs and to develop mechanisms for showing positive appraisals for volunteer CHWs. This study contributes stronger evidence with regard to retention than the earlier study we conducted, because we employed a prospective cohort study design. Few researchers in studies of retention have used a similar methodology, and the use of more robust designs such as ours may better contribute to identifying long-term strategies that can be used to increase the level of retention and ensure sustainability of volunteer CHW programs.

## Competing interests

The authors declare that they have no competing interests.

## Authors’ contributions

Both KA and EO conceptualized the study design and protocol. KA led the project as Principal Investigator, analysed the data and wrote the manuscript draft in consultation with EO. Both KA and EO finalized the manuscript. Both authors read and approved the final manuscript.
